# Comparison of hybridization-based and sequencing-based gene expression technologies on biological replicates

**DOI:** 10.1186/1471-2164-8-153

**Published:** 2007-06-07

**Authors:** Fang Liu, Tor-Kristian Jenssen, Jeff Trimarchi, Claudio Punzo, Connie L Cepko, Lucila Ohno-Machado, Eivind Hovig, Winston Patrick Kuo

**Affiliations:** 1 Department of Tumor Biology, Rikshopitalet-Radiumhospitalet Medical Center, Montebello, NO-0310 Oslo, Norway; 2 Department of Medical Informatics, Rikshopitalet-Radiumhospitalet Medical Center, Montebello, NO-0310 Oslo, Norway; 3 PubGene AS, Vinderen, NO-0319 Oslo, Norway; 4 Department of Genetics, Harvard Medical School, Boston, MA, USA; 5 Howard Hughes Medical Institute, Harvard Medical School, Boston, MA, USA; 6 Decision Systems Group, Brigham and Women's Hospital, Boston, MA, USA; 7 Department of Developmental Biology, Harvard School of Dental Medicine, Boston, MA, USA; 8 Department of Organismic and Evolutionary Biology/Faculty of Arts and Sciences, Harvard University, Cambridge, MA, USA

## Abstract

**Background:**

High-throughput systems for gene expression profiling have been developed and have matured rapidly through the past decade. Broadly, these can be divided into two categories: hybridization-based and sequencing-based approaches. With data from different technologies being accumulated, concerns and challenges are raised about the level of agreement across technologies. As part of an ongoing large-scale cross-platform data comparison framework, we report here a comparison based on identical samples between one-dye DNA microarray platforms and MPSS (Massively Parallel Signature Sequencing).

**Results:**

The DNA microarray platforms generally provided highly correlated data, while moderate correlations between microarrays and MPSS were obtained. Disagreements between the two types of technologies can be attributed to limitations inherent to both technologies. The variation found between pooled biological replicates underlines the importance of exercising caution in identification of differential expression, especially for the purposes of biomarker discovery.

**Conclusion:**

Based on different principles, hybridization-based and sequencing-based technologies should be considered complementary to each other, rather than competitive alternatives for measuring gene expression, and currently, both are important tools for transcriptome profiling.

## Background

During the last decade, a considerable number of high-throughput technologies for transcriptome profiling have been developed. These include hybridization-based technologies, such as DNA microarrays [1-3], and sequencing-based approaches like SAGE (Serial Analysis of Gene Expression) [4] and MPSS (Massively Parallel Signature Sequencing) [5]. The power of DNA microarrays lies in the simultaneous hybridization of mRNA extract from biological samples to a pre-selected mRNA library, which can contain up to tens of thousands of various mRNA transcripts. The expression levels of each transcript are obtained by reading out intensities of hybridization signals. Sequencing-based methods are based on a substantially different strategy as compared to microarray technologies. SAGE and MPSS do not require any pre-compilation of an mRNA library of sequences, but instead, they use type IIS restriction endonucleases, i.e. tagging enzymes, to collect short tags (typically 10–22 bases) from each mRNA molecule, provided a relevant recognition site exists for an anchoring enzyme. Then, either by sequencing long concatamers of tags using conventional sequencer (SAGE), or by performing iterative parallel sequencing using a proprietary technique (MPSS), the identity of a sufficiently large amount of tags can be determined in an efficient manner. The abundance of each mRNA transcript is assumed to be proportional to the count of occurrence of its representative tag.

Favorable features of hybridization-based approaches include a significantly lower workload and relatively low cost. However, the probe collection on a chip, which necessarily relies on the coverage and the accuracy of both genomic sequences and clone libraries, presents a hard constraint on its detection power. In contrast, the "tag-and-count"-based methodologies require more advanced instruments that are more cost- and labor-intensive, but their capability of exhaustive transcript sampling allows the potential identification of novel mRNAs.

The present co-existence of various DNA microarray platforms and sequencing-based technologies offers biomedical research increased options for transcriptome profiling. It is, however, important to understand how to compare data generated by these different technologies. Recent studies indicate that performance of various microarray platforms, as measured by data consistency, have been shown to be comparable [6,7]. Several attempts have also been made to compare heterogeneous types of technologies, for instance, between microarray and SAGE [8-15], and between microarray(s) and MPSS [16-20]. The results of these studies demonstrate moderate concordance between technologies.

In the present study, which is part of an ongoing cross-platform comparison framework [6], we compare gene expression data from MPSS with data from five different commercially available one-dye microarray platforms. The present study extends our previous study in three ways: 1) the inclusion of the Illumina BeadArray^® ^and MPSS data, 2) the inclusion of gene expression data of a second pool of mouse retina (MRP2) for all microarray platforms, and 3) the investigation of variation across biological replicates, as measured over two different pools of mouse retina samples (MRP1 and MRP2). It has been a consensus that the use of biological replicates is an important element to ensure the reliability of microarray results [21]. To our knowledge, this is the first study investigating the differences between microarray and MPSS data on biological replicates. MPSS libraries are usually constructed without technical replication and data variation across samples are generally estimated by applying a statistical model simulating the random sampling process during tag selection for sequencing [22]. It remains unclear whether significant variation across samples examined by MPSS is comparable with those detected by microarrays. As our study included technical replicates for the microarray platforms, this provided a unique opportunity to investigate the sampling model by comparison to microarray data where technical replicates permitted a more robust statistical testing. The data sets of microarray data for the first mouse retina pool (MRP1) and mouse cortex (MC) were analyzed in our previous study, but some results on these are also included here for the sake of performing comparisons between the two mouse retina pools, as we believe the investigation of biological replicates is an important and novel aspect of this study.

In summary, our results showed that there were moderate, yet significant, correlations between microarray data and MPSS data, while the data from microarray platforms, including the recently included Illumina arrays, generally were well correlated. The majority of discrepant measurements between the technologies based on hybridization versus sequencing were genes with low-abundance transcripts. Tag-to-gene mapping ambiguity and the absence of tagging enzyme recognition site could also explain some of the discrepancy between MPSS and microarrays. Using two-way ANOVA and SAM, we examined the microarray data for the magnitude of data variation introduced by biological replicates and technical replicates, and showed that biological variations are smaller than platform variations. The genes we found most susceptible to variations between biological replicates were likely to be associated with metabolic pathways, biosynthesis, and binding related pathways. Due to their vulnerability to sample variation, any changes observed in these pathways and related genes should be interpreted more cautiously in biomarker discovery applications. Furthermore, as demonstrated in our study, the relative affordability of array replicates makes them useful to corroborate MPSS experiments where technical replicates are seldom feasible due to higher costs. For comprehensive transcriptome profiling, we suggest that complementary use of hybridization-based and sequencing-based technologies is likely to provide a better solution than pursuing a single type of technology alone.

## Results

### Characterization of MPSS data

MPSS libraries for MRP1 and MRP2 were generated using 'Signature' MPSS protocol. Two alternative sequencing reactions conducted independently, i.e. two-stepper and four-stepper sequencing, provided two read-outs of tag sequences for each sample, and referred to as MPSS 17-bp and MPSS 20-bp, respectively.

In the 17-bp signature sequencing, a total number of 34,341 signatures were detected for MRP1, and 29,509 signatures for MRP2. The total number of signatures was obtained in the 20-bp signature sequencing was 34,424 and 30,967, for MRP1 and MRP2, respectively.

The assignments of signature to gene were performed as described in the Methods section. Only the reliable MPSS signatures were kept in the downstream analysis. In the 17-bp signature libraries, 6,001 and 5,340 unique UniGene identifiers for MRP1 and MRP2, respectively, were identified. This corresponds to the sum of transcript copies as 615,944 and 615,771 tpm (transcript per million). For the 20-bp tag collections, the numbers of unique UniGene identifiers were slightly smaller: 5,793 and 5,125 for MRP1 and MRP2, respectively, which corresponds to the sum of transcript copies as 655,919 and 652,588 tpm.

### Characterization of microarray data

All platforms showed generally good consistency among technical replicates for all samples, both in terms of CVs and correlation coefficients (see Additional file 1). These two metrics did not reveal noticeable differences between the two mouse retina mRNA pools within each platform.

In order to compare data from microarray platforms, we calculated both Pearson and Spearman correlation coefficients for absolute expression levels and relative expression changes. To transform expression measurements from diverse platforms onto a common scale, percentile transformation was applied to absolute expressions for each array data set, and then the median of percentile transformed intensities across replicated measurements per gene was used (see Methods for details). Filtering was applied but was not observed to increase the correlations between intensities, while the correlations between log_2_ratios were considerably improved with filtering.

The inter-platform data agreement by measuring correlation coefficients is shown in Additional file 1. The correlations between Illumina and the other platforms were generally lower than between other pairs of microarray platforms. This could possibly be due to the lack of technical replicates for Illumina.

### Comparison between microarrays and MPSS

#### Statistics of overlapped genes between microarray and MPSS

Table 1 provides a summary of the overlap of genes (UniGene clusters) expressed in MPSS and the microarray platforms. In each pool, MPSS was found to have similar numbers of overlapping genes with all microarray platforms, with Illumina having the smallest number of common genes. This fact contradicted our knowledge that Illumina as well as ABI have the newest probe collections and higher probe densities. This may have been because these newer chips had to be mapped to an older UniGene build in order to use the same build as the ones used for the other platforms that were already included earlier in the study. Generally, MPSS from MRP1 presented a larger number of overlapping genes with the microarrays than MPSS from MRP2.

**Table 1:** Statistics on overlapping genes between microarray platforms and MPSS libraries.

***MRP1***								
***Affymetrix***	***Amersham***	***Mergen***	***ABI***	***Illumina***	***MPSS (17-bp)***	***MPSS (20-bp)***		

**Affymetrix**	5896	5777	3584	2836	2250	1981	3021	2685
**Amersham**	3582	7311	7525	3693	2963	2528	3263	2832
**Mergen**	2785	3469	4813	5032	2140	1803	2368	2078
**ABI**	2299	2870	2041	11429	11418	6725	2467	2092
**Illumina**	2003	2470	1739	6716	11074	11071	1950	1878
**MPSS (17-bp)**	2813	2923	2101	2190	4439	2192	4962	4688
**MPSS (20-bp)**	2718	2807	2026	2097	4172	2141	4261	4808

	**Affymetrix**	**Amersham**	**Mergen**	**ABI**	**Illumina**	**MPSS (17-bp)**	**MPSS (20-bp)**	

**MRP2**								

Expression profiles of the two mouse retina pools (MRP1 and MRP2) were obtained on five one-dye microarray platforms (Affymetrix, Amersham, Mergen, ABI, Illumina), as well as MPSS technology. Genes were matched based on mapping to UniGene cluster IDs. For each pair of data sets, the number of common genes with expression data after filtering is shown. The upper triangle and lower triangle represent the statistics for MRP1 and MRP2, respectively. The upper (right-most) and lower (left-most) diagonals show the total number of genes after filtering for each data set for MRP1 and MRP2, respectively.

#### Correspondence between microarray and MPSS

The Pearson correlation coefficients of log10-transformed gene expression were used to measure the overall linearity between microarray and MPSS data, since it is well known gene expression data often have a distribution close to log-normal. As shown in Table 2, the cross-technology data correlations, i.e. between hybridization-based data and sequencing-based data, were found to be poorer than those of within-technology comparison. Taking MPSS 17-bp signature data as an example, the Pearson correlations of the logarithmic transformed expressions between MPSS data and microarrays ranged from 0.39 (MPSS – Illumina) ~0.48 (MPSS – Affymetrix) for MRP1, and 0.40 (MPSS – Illumina) ~0.52 (MPSS – Affymetrix) for MRP2. It is expected that a 17-bp signature decoding would result in higher sensitivity, while the 20-bp one would provide better specificity, but it is unclear whether they would differ in terms of their correlation with other platforms. From our analyses, we observed that MPSS 17-bp and 20-bp tag lengths have comparable correlations to microarray platforms, as shown in Table 2.

**Table 2:** Pearson correlation coefficients across data sets

	**Affymetrix**	**Amersham**	**Mergen**	**ABI**	**Illumina**	**MPSS (17 bp)**	**MPSS (20 bp)**
**Affymetrix**	**0.99**	0.58	0.49	0.53	0.49	0.48	0.47
**Amersham**	0.56	**0.99**	0.51	0.60	0.52	0.45	0.45
**Mergen**	0.49	0.50	**0.99**	0.48	0.42	0.41	0.39
**ABI**	0.53	0.59	0.46	**0.99**	0.56	0.41	0.40
**Illumina**	0.51	0.51	0.44	0.55	**0.99**	0.39	0.38
**MPSS (17 bp)**	0.52	0.45	0.44	0.43	0.40	**0.65**	0.98
**MPSS (20 bp)**	0.51	0.46	0.44	0.44	0.40	0.98	**0.65**

Gene expression data after filtering (microarray intensities and MPSS tag counts) were log_10_-transformed prior to calculating the Pearson correlations. Both microarray and MPSS measurements were mapped to UniGene clusters. The upper and lower triangle shows pair-wise correlations for data on MRP1 and MRP2, respectively. The numbers on the diagonal are the within-platform correlations between MRP1 and MRP2.

**Figure 1:**
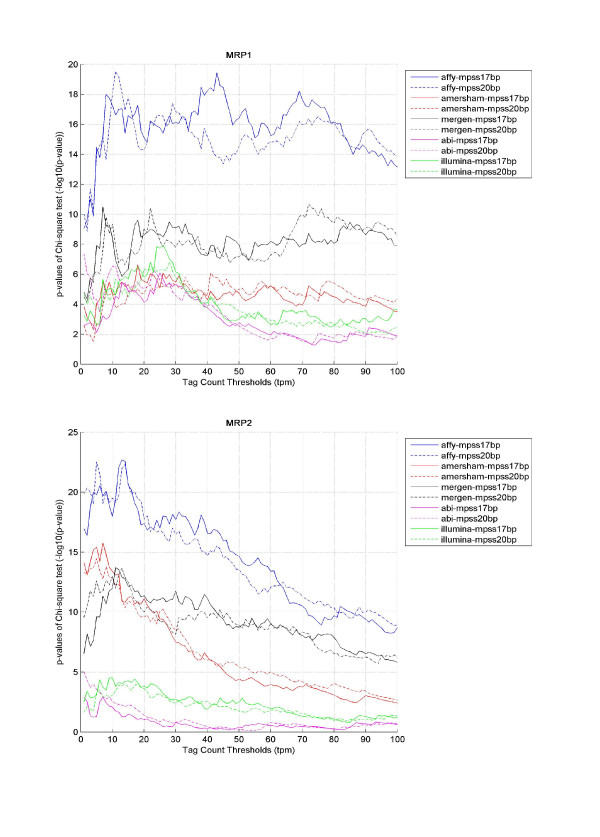
**Correspondence of expression detection between microarrays and MPSS**. Chi-square statistics was used as a measure of data correspondence in terms of absent/present call made by each microarray platform versus each MPSS library. For microarray data, a fixed definition of the "Present/Absent" call based on quality flags and filtering status for each gene. For MPSS data, a gene (UniGene clusters) was considered "Present" if the copy number was above a given threshold, otherwise it was considered "Absent". The "Present/Absent" call threshold varied from 1 to 100 tpm for MPSS in steps of 1 and Chi-square statistics were calculated for each threshold. Part (a) shows how the p-values from these comparisons varied with threshold on data for MRP1, and part (b) shows the same for MRP2.

**Figure 2:**
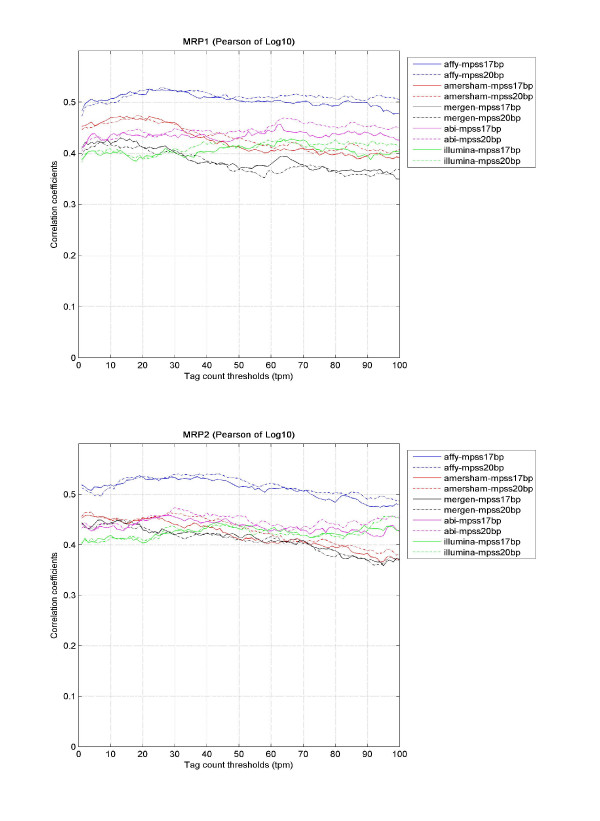
**Correspondence of expression level between microarrays and MPSS**. Pearson correlation was used as a measure of correspondence of expression levels reported by each microarray platform versus each MPSS library. For microarray data, normalized and filtered intensities were used. With the tag count threshold being varied from 1 to 100 tpm for MPSS in steps of 1, Pearson correlations were calculated for the genes with tag counts above each given threshold. Part (a) shows how the correlations from these comparisons varied with threshold on data for MRP1, and part (b) shows the same for MRP2.

Next, we wanted to examine how data correspondence, both in terms of binary presence call and correlation of expression, across the two technologies varied as a function of transcript abundance. In MPSS, a gene was defined as present if the copy number was higher than a given tag count threshold, otherwise, it was considered absent. These calls were combined with microarray present/absence calls to perform a chi-square test to measure the agreement of present and absent calls between the technologies. When the threshold of tag count changed from 1 to 100 at a step of 1 (tpm), we obtained the binary call agreements as a function of tag abundances (see Figure 1). We observed that the technologies often have the best correspondence when copy number threshold was set around 8~12. As the threshold increases, the correspondence measure tended to drop, which probably can be attributed to the drastically reduced number of MPSS measurements above the given threshold. The trend of data correspondence versus tag count thresholds for each sample was similar among all microarray platforms. Similarly, we also obtained data correlations when applying different tag count thresholds (see Figure 2). The fluctuations in data correlations were small, but overall, Affymetrix had better presence-call agreement and linear correlations with MPSS than the other microarray platforms.

**Figure 3:**
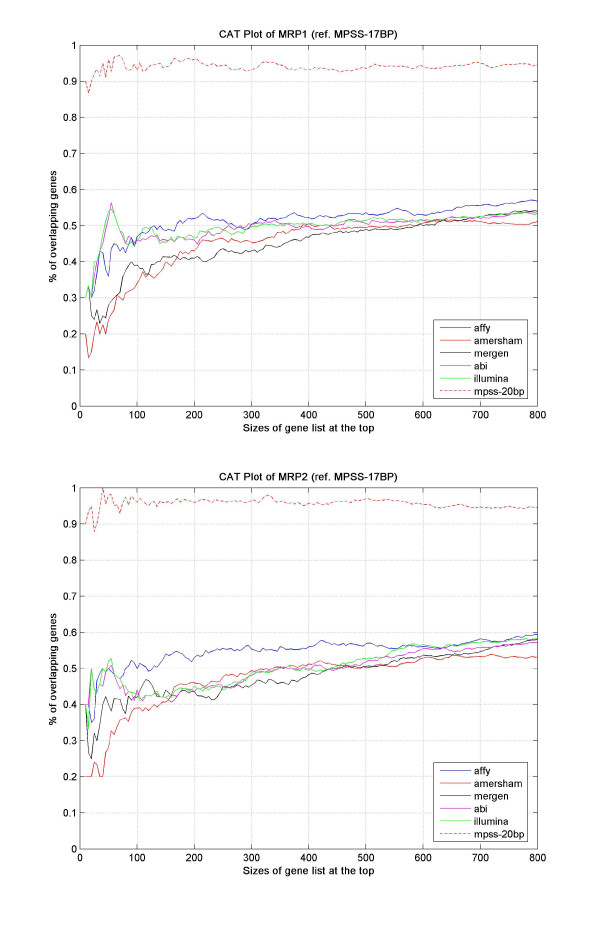
**CAT plots using MPSS data as reference**. Correspondence between MPSS and microarray platforms on detection of highly expressed genes was assessed by CAT plots. In Figure 3, the x-axis represents the sizes of gene sets at the top of gene expressions, from 10 to 800 at a step of 5, and the y-axis shows the percentage of overlapping genes between each microarray platform and MPSS in the given set of genes. Part (a) represents MRP1, and part (b) shows MRP2.

We also investigated the relationships for highly abundant genes. Based on the CAT (Correspondence at The Top) plots [23] (see Figure 3 and Additional file 2), we observed that Illumina, ABI and Affymetrix had higher correspondence at the high-abundance gene expression levels, whereas Amersham had the lowest. For all platforms, the correspondence with MPSS gradually improved and converged when the gene set at the top was increased.

Furthermore, we found that 305 genes (286 in MRP1 and 272 in MRP2) were present across five microarray platforms, but were not detected by MPSS, neither from the 17-bp library nor the 20-bp library. For both libraries, about 64% of these genes were filtered out in the tag-to-gene mapping procedure. A total of 97 genes were not detected by MPSS at all (any sample or library), among which 11 were labeled "GATC"-negative. Using percentile-transformed data, we found that 67.8% of the remaining genes (60 of 86) were detected as having low expressions (percentile less than or equal to 50). This indicates that low-end sensitivity is the major reason for data discrepancy.

Taking the whole MPSS libraries as gold standard, we defined false negative detections by each microarray as the number of genes that were detected by MPSS, as well as printed on the chip, but were not detected by the microarray. We found that the proportion of false negatives in the five microarray platforms ranged from 12.99% (Affymetrix, MRP1) to 2.18% (ABI, MRP1).

### Evaluation of data variations in biological replicates

#### Using SAM to characterize variation in detection of expression changes across biological replicates

**Figure 4:**
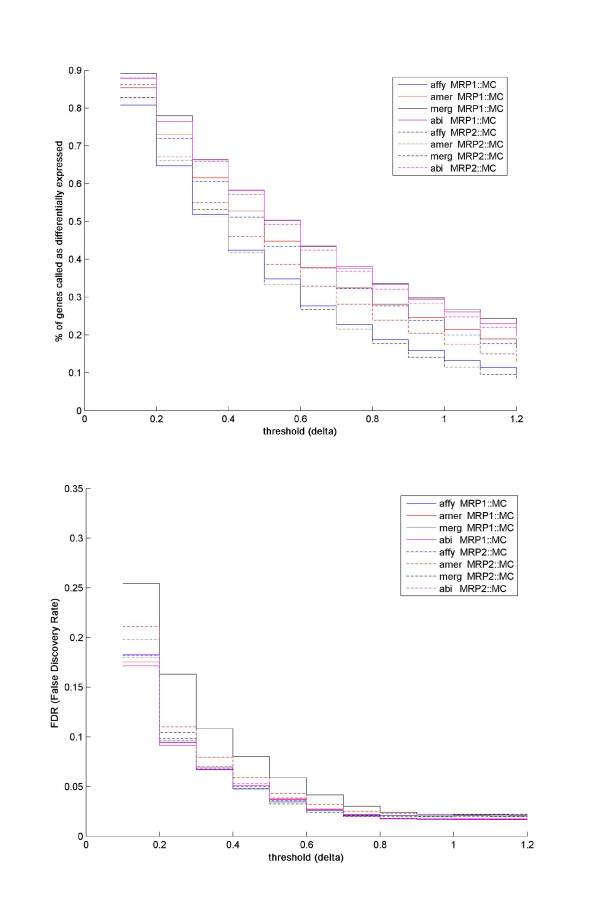
**Statistics of differentially expressed genes identified by SAM as a function of "delta" threshold**. In Figure 4, the x-axis represents the threshold ("delta") for differential expression identification which changes from 0.1 to 1.2 at a step of 0.1, while the y-axis is in (a) the percentage of genes which were considered to be differentially expressed at a given threshold; and in (b) false discovery rate.

Based on the data from the microarrays where we had technical replicates, we used SAM to compare the detection of differential expression from MRP1::MC versus MRP2::MC, i.e., differential expression was investigated between the two mouse retina replicates based on a common reference of mouse cortex. As shown in Figure 4, the experiment groups across platforms and biological replicates, except for MRP1::MC experiments on Mergen, had quite comparable performance with respect to FDR (False Discovery Rate) versus delta. Delta represents the cutoff outside which genes are identified as differentially expressed by having a larger modified t-statistic than expected under the null model (a low delta value normally gives a high FDR, and vice versa). All platforms, except for Affymetrix, showed a larger number of differentially expressed genes in MRP1::MC than in MRP2::MC. The two retina pools showed the most disparate behavior on the Mergen platform, and the least on the ABI platform. Moreover, when looking at MRP1::MC and MRP2::MC combined, ABI tended to call a higher percentage of genes as differentially expressed between mouse retina and cortex samples than the other three microarray platforms.

#### Two-way ANOVA to identify individual genes with significant variation

Two-way ANOVA analysis was performed to identify the contribution of platforms and samples on data consistency. We examined filtered log_2_ratios (MRP1::MC and MRP2::MC) after normalization matched by both RS (RefSeq) and RSEXON (RefSeq ID and exon) across all four platforms where technical replicates were available. Only those genes that did not have missing values across the 10 measurements were used in this analysis. In RS- and RSEXON-based matching, 563 and 305 genes, respectively, satisfied the criterion. The threshold of statistical significance was chosen as p < 0.001.

The majority of genes did not exhibit any bias induced by neither sample replicate nor by platform. For the two matching options 52.0 % (293 of 563) and 55.9% (170 of 304) of the genes for RS-based and RSEXON-based matching, respectively, were robust both in sample and platform variability. Based on Table 3, it can be seen that the number of genes that were subject to platform-induced variability was larger than the sample-induced variability. The data in this table also further support that probes matched at the exon level (RSEXON-based matching) across platforms resulted in lower data variations than those matched at the transcript level (RS-based matching), as previously observed [6].

**Table 3:** Summary of two-way ANOVA results

	**RS**	**RSEXON**
total # of genes (i.e. RSs or RSEXONs)	563	304
# of genes that showed neither sample- nor platform- related variation	293	170
# of genes that showed sample-related variation	27	13
# of genes that showed platform-related variation	260	132
# of genes that showed both sample- and platform- related variation	20	11

Two-way ANOVA was conducted for all microarray platforms that had been analyzed with technical replicates (Affymetrix, Amersham, Mergen, ABI). The genes that have valid measurements after normalization and filtering procedure in all technical replicates were included for the analysis. The analysis was conducted on gene expression measurements matched across platforms based on RefSeq ID (RS) and RefSeq Exon (RSEXON). A gene was said to have sample-related variation if the p-value of the sample effect size was significant (p < 0.001), and similarly for platform-related variation.

#### Comparison of data variation between microarray and MPSS

From the four microarray platforms with technical replicates, we used one-way ANOVA to identify genes with differential expression in MRP1 versus MRP2. These results were then compared with the Z-test statistic commonly used for detecting differential expression in MPSS data. The microarray data used for this analysis was restricted to genes that had valid measurements for all ten technical replicates. We also excluded measurements from probes with ambiguous UniGene mapping, which was used to match microarray data with MPSS data. For the four microarray platforms, Affymetrix, Amersham, Mergen, and ABI, we obtained 4,783, 5,987, 3,610, and 8,460 UniGene identifiers with corresponding p-values from one-way ANOVA, respectively.

Taking a threshold p-value of 0.001, we summarized the concordant and discordant calls between the microarray one-way ANOVA test and the MPSS Z-test, as represented in Table 4. The proportion of concordant calls between microarrays and MPSS were very similar among various platforms and 17-bp/20-bp MPSS signatures, ranging from 60.04% ~63.43%. Most of the discordances between microarrays and MPSS were cases were differential expression was detected by MPSS but not by microarrays: ~94% for Affymetrix, ~89% for Amersham, ~79% for ABI, and ~97% for Mergen. Data variation between biological replicates with MPSS without technical replicates may understandably be larger than with microarrays when using sufficient numbers of replicates. It is easier to obtain robust statistical testing based on replicates. Also in this analysis, we observed that the statistics with MPSS 17-bp results and the 20-bp results did not differ significantly, consistent with the results of previous analyses.

**Table 4:** Comparison of differentially expressed gene identification between microarrays and MPSS

	**Affymetrix**		**Amersham**		**Mergen**		**ABI**	
	
	**MPSS (17-bp)**	**MPSS (20-bp)**	**MPSS (17-bp)**	**MPSS (20-bp)**	**MPSS (17-bp)**	**MPSS (20-bp)**	**MPSS (17-bp)**	**MPSS (20-bp)**
**Number of genes included in the comparison**	1912	1827	2248	2127	1518	1452	1628	1535
**Number of genes considered as differentially expressed in both microarray and MPSS**	29	30	49	46	17	16	81	79
**Number of genes considered as having NO differential expression in both microarray and MPSS**	1119	1084	1350	1277	937	905	900	858
**Number of genes that are considered as differentially expressed in MPSS but not in the microarray**	714	664	761	717	550	517	514	473
**Number of genes that are considered as differentially expressed the microarray but not in MPSS**	50	49	88	87	14	14	133	125

One-way ANOVA was conducted for all microarray platforms that had been analyzed with technical replicates (Affymetrix, Amersham, Mergen, ABI) to identify differentially expressed genes between samples, MRP1 and MRP2. For MPSS libraries, a Z-test was used to identify differentially expressed genes, since technical replicates were not available. A threshold p-value of 0.001 was used for both statistical tests to define differential expression. Based on UniGene cluster mapping, identification of differentially expressed genes from MPSS was compared to identification of differentially expressed genes from the microarrays. For each microarray platform, a column corresponding to comparison with the 17-bp tag library and a column corresponding to the 20-bp tag library are shown. The detailed comparisons of agreement/disagreement were restricted to the common UniGene clusters where there was a one-to-one correspondence with MPSS tags.

#### GSEA to summarize differences between biological replicates using biological themes

For each microarray platform, we applied GSEA to generate hypotheses regarding which biological processes or pathways might be responsible for the differences between the two replicate sample pools. Only the genes (RSs) which had valid values across all 10 chips were used, that is, 2,490 for Affymetrix, 4,899 for Amersham, 3,132 for Mergen, and 7,556 for ABI. In GSEA, the GO hierarchy level was set to 4, and the permutation times as 1000. Several biological themes resulted from GSEA with enrichment score, but none were statistically significant. Although different microarray platforms revealed different degrees of variation between MRP1 and MRP2, the GSEA analyses on the affected gene sets were similar among platforms. When considering enriched themes found in all four platforms, there were 15 GO terms from the "biological process" category enriched in MRP1 compared to MRP2 and 12 GO terms from the "molecular function" category. There were no terms found to be enriched in MRP2 common to all platforms [see Additional file 3].

## Discussion

With microarray technology being rapidly developed and advanced in the past decade, it has become an important tool for studying gene expression patterns. In parallel, the technologies based on the "tag-and-count" principle, such as SAGE and MPSS, are also being used for exploring the full transcriptome. Although some efforts in designing and adopting standards of microarray experiments (e.g. ERCC [24]) and data deposition (e.g. MIAME [25]) are paving the way towards data meta-analyses and integration, it remains a critical challenge to systematically compare cross-sample, cross-platform, and cross-technology data. To this end, we have established a framework which accommodates various platforms and various technologies, using quality-controlled biological samples [6]. Several recently published studies [7,23,26] have concluded that, for DNA microarray technology, the reproducibility of technical replicates both within a given platform and across platforms are generally good, especially when the experimental design, protocols, and data analyses are standardized [6]. In this study, we have examined data correspondence and discrepancy between MPSS and one-dye microarray platforms, representing sequencing-based and hybridization-based technologies, respectively. We examined whether and how the behavior of these distinct technologies would vary when challenged with biological replicates.

The main observations made in this study were that across the microarray platforms, both intra- and inter-consistency of data was generally high, but the agreement between MPSS and any microarray platform was moderate, as also reported in previous studies [17,18]. The differences in signal detection between MPSS and microarray platforms were observed both in terms of lower correlations and in terms of genes that were consistently found as expressed in MPSS but not in microarrays and vice versa. These findings were also identified in a similar study [18], where this could be the reflection of the known limitations of the MPSS technology [27]. Although mapping problems may have contributed to the observed discrepancies, it is more likely that inherent differences between hybridization-based and sequencing-based technologies caused the systematic differences in gene expression detection between MPSS and microarrays. For a given microarray design, the set of genes that can be detected is pre-determined, while for MPSS and similar technologies, the main limitation is that presence of a recognition site is a requirement for detection. However, when comparing the genes represented on the various microarrays to the genes detected as expressed with MPSS, we found approximately 80% of the genes on the ABI and Illumina arrays had not been found in the MPSS libraries and about half of the genes for the Affymetrix, Amersham and Mergen arrays. This suggests that all the microarray designs were comprehensive with respect to genome coverage, and that the fixed probe sets may not have been a main limitation in this study. Furthermore, in all microarray platforms there were a number of genes that were detected as present but not found in the MPSS libraries. Among the 97 such genes (UniGene IDs) common to all five microarray platforms, there were 11 that were found to be lacking the DpnII recognition site ("GATC") and could not be expected to give any signal in MPSS. For the remaining 86 genes, the expression measurements on the microarrays varied and included genes classified as consistently highly expressed, as well as genes classified as having consistent low expression. An investigation of the probe sequences of the most highly expressed genes in the microarray platforms did not reveal noticeable differences in the number of possible sequence matches between those found in MPSS and those not found in MPSS. It is nevertheless possible that some of the false-positives in microarrays relative to MPSS could be caused by cross-hybridization due to suboptimal probe design. However, without a gold-standard, it is not possible to ascertain that the microarrays are overestimating the expression for these genes. For the genes identified as having lower expression in microarrays but not found in MPSS, it is possible that this may have been caused by running the MPSS experiments with insufficient sampling depths resulting in a less representative sampling of tags. There were also a number of genes that can be regarded as "false-negatives" on the microarrays relative to MPSS, in the sense that they were represented on the microarrays and detected by MPSS but not detected as expressed by the microarrays. For these genes there was less consistency across the platforms and only one gene detected by MPSS was not identified as expressed by any of the microarrays where it had been represented. Again, suboptimal probe design due to incomplete sequence knowledge can be a factor. Other possible reasons include MPSS sequencing errors [19], high complexity in transcriptional activities [28], heterogeneity in polyadenylation cleavage sites [29] and various sequence-introduced biases [30,31]. We have however not been able to estimate the size of such contributions in this study. It has also been reported that the existence of SNPs [32] can influence the interpretation of digital-based experimental data such as MPSS or SAGE, but this is not expected to have been a major contributing factor in this study. The fact that the mapping to UniGene IDs had been based on two different versions of the UniGene database is a possible confounding factor. However, this cannot explain all the discrepancies as several genes were manually checked against the latest UniGene build and found to have consistent mappings.

Data filtering is commonly applied both to microarray and MPSS data. Both in the present study and an earlier study, we have shown that data filtering considerably improves data consistency between microarray platforms, and in particular on relative expression (log_2_ratios). The low intensity signals generally corresponding to low-abundance transcripts are typically filtered as it is expected that the signal-to-noise ratio becomes too small. The comparisons between MPSS and microarrays indicate that the MPSS technology also has problems in detecting low-abundance transcripts. It appears that neither technology can reliably detect transcripts expressed at very low frequencies in an environment where the expression levels of all transcripts could span several orders of magnitude. This is a major problem as many transcripts have low-abundance, and there is currently a consensus that many of the corresponding genes are associated with critical regulatory roles in the cells [33]. For microarrays to improve the low-end sensitivity, the optimization of probe design, as well as the advances of scanning techniques, may be key issues. In sequencing-based systems, increase of library size and sequencing accuracy may increase the confidence in low-abundance transcripts identification.

Recent studies have demonstrated that factors such as strain [34,35], gender [36,37], as well as diet [38,39] and circadian variation, [40] can influence gene expressions in various organisms and tissues. In our study we created two retina samples by randomized pooling of samples from a large number of individuals aiming to cancel out the effects of such factors. We examined the biological variability at several levels: (1) the influence of biological replicates on the measures of intra- and inter-platform data consistency; (2) the overall capability of differentially expressed gene identification by each biological replicate; and (3) the genes or gene sets that were most susceptible to biological variability between MRP1 and MRP2. In general, the two pools showed very similar gene expression patterns as expected, but for some genes there was a difference between the two mouse retina pools that was consistent across the platforms and technologies. Moreover, MPSS also differed considerably from the microarray platforms in terms of identification of genes differentially expressed in MRP1 versus MRP2. As is commonly done due to high instrumental complexity and cost, MPSS data for each sample was collected without technical replicates, and differentially expressed genes were identified by a statistical approach. The Z-test, found by Man *et al*. [22] to perform well in terms of specificity, power, and robustness for determining statistical significance in SAGE, detected far more differentially expressed genes than the statistical tests applied to the microarray data with technical replicates. Also in terms of fold-change, MPSS detected far more genes as having two-fold or larger change than the microarray platforms. In this respect, Illumina data, which also did not have technical replicates, behaved similarly as the other microarray platforms, hence the lack of technical replicates alone cannot explain all of these differences. In light of the construction of the two retina pools and the good agreement between the microarray platforms, this may be a sign of caution for those who intend to use MPSS for the purpose of biomarker discovery without incorporating technical replicates. Until technological advances make technical replicates in MPSS feasible and MPSS data are further studied and confirmed using independent and complementary technologies, MPSS may not be the optimal choice for identification of novel fingerprints based on differential expression.

The GSEA results across the microarray platforms with technical replicates indicated some common biological themes for the differences between MRP1 and MRP2. Although the results were not statistically significant, they were very consistent across the platforms and indicated metabolism and transcriptional regulation as general biological themes describing the differences. This was also confirmed by GOstat [41] analysis using a list of genes consistently identified as differentially expressed when also including MPSS data (data not shown). Based on the construction of the two retina pools, these results indicate that caution should be exercised when interpreting results of differential expression.

An earlier study [42], which compared microarray data, EST-based expression experimental data and SAGE using published data sets, reached the conclusion that the agreement between the methods was highly variable from gene to gene, and the authors advocated the need for gene-by-gene validation of important global gene expression measurements using non-global methods. The present results support a similar conclusion, and we emphasize that sequencing-based methods in general as well as hybridization-based methods have inherent technological limitations. Microarrays are limited by pre-selected gene sets and possible cross-hybridization problems, as was indicated in this study. Apart from the obvious limitations of restriction site presence, MPSS is an open-ended system but has problems related to the mapping of tags limiting the set of genes for which it is possible to obtain reliable measurements. Altogether, this suggests that exploitation of the complementarity of these technologies is a better approach for global transcriptome analysis.

## Conclusion

Overall, the agreement between MPSS and microarrays was significant, but lower than between different microarray platforms. Measurements of genes with low expression more often disagreed than highly expressed genes, as expected, but also for genes with high expression there were systematic differences in detection. We found that differences in gene expression measurements between MPSS and microarrays are not only due to increased sensitivity of MPSS to low abundance transcripts and the ability of MPSS to measure new transcripts. Further studies comparing sequencing-based and hybridization-based technologies, including both biological replicates using different types of tissue samples as well as technical replicates are warranted in order to delineate in more detail the shortcomings of these technologies. Future methodological development will be necessary to maximize the information derived from the two complementary types of technology.

## Methods

### Biological samples

RNA samples were isolated from three sources: two pools of C57/B6 adult mouse retina (MRP1 and MRP2, n = 700) and Swiss-Webster post-natal day one (P1) mouse cortex (MC) (n = 19). Retinas were dissected, collected and stored in Trizol (one pair of retinas per eppendorf tube) at -80°C prior to pooling. During the RNA extraction process, two pools of adult mouse retina (MRP1, MRP2) were created (700 retinas per pool), aliquoted. All samples were stored at -80°C until being used in experiments. The animal experiments were approved by the Institutional Animal Care Facility at Harvard University.

### Microarray platforms, data processing and consistency assessment

Whole-genome mouse gene expression arrays (one-dye oligonucleotide microarrays) were investigated in this study, including: Affymetrix GeneChip^®^, Amersham (now GE Healthcare) CodeLink^®^, Mergen ExpressChip^®^, Applied Biosystems (ABI) microarrays, and Illumina BeadArray^®^. Microarray experiments are composed of sample preparation, hybridization, scanning and image quantitation, which are a series of integrative procedures being conducted at a laboratory, generally according to the manufacturer's recommended protocols. To obtain sufficient statistical confidence in the data analysis, for each biological replicate (MRP1 and MRP2), five technical replicates on each platform were obtained, with an exception on Illumina. We wanted to include Illumina in this study to examine the magnitude of data variation in MPSS experiments and microarray experiments when no technical replicates are performed. For details of the experimental protocols and laboratories, we refer to Kuo *et al*., [6] except for Illumina, which can be found in the Additional file 4.

The raw data sets of 63 chips after image scanning and quantification in each platform were collected. For Illumina data, we set the filtering threshold as "Detections" = 0.9. Filtering for the other microarray platforms are described in Kuo *et al*. [6]. We also performed percentile transformation of intensities, quantiles normalization and log_2_ratio calculation, as described.

Data repeatability and reproducibility [7] are two important aspects of microarray data consistency assessment. In this study, the former will refer to the degree of data variations among technical replicates of a platform, and the latter will refer to data agreement across different microarray platforms when using the same biological samples. Two popularly used metics, coefficient of variations (CV) among replicated measurements per gene and correlation coefficient (Pearson and Spearman correlations) between any pair of replicated experiments, were adopted to assess repeatability and reproducibility. For intra-platform data consistency, the mean and standard deviation of CVs or correlation coefficients were used as summations of each platform's performance. For inter-platform data agreement, either the mean (for normalized log_2_ratios) or the median after percentile transformation (for intensities) of repeated measurements on each platform were used in calculating correlation coefficients.

### MPSS experiment and data processing

Total RNA of MRP1 and MRP2, which were identical to those used in microarray experiments, was sent to Lynx Therapeutics, Inc. (now Illumina, Hayward, CA) for 'Signature'-based MPSS experiments. Following an RNA quality test on a Agilent 2100 BioAnalyzer (Agilent Technologies, Palo Alto, CA), cDNA libraries were generated according to the Megaclone protocol [5,43]. Signatures adjacent to poly (A) proximal DpnII restriction sites ("GATC") were cloned into a Megaclone vector. The resulting library was amplified and yielded about 1.6 million loaded microbeads, which were loaded onto a flow cell. Thereafter, an iterative series of enzymatic reactions decoded the signatures as 17-bp or 20-bp sequences (including DpnII recognition sites "GATC") [44].

The abundance of each signature was converted to transcripts per million (tpm), and the MPSS signatures were mapped to UniGene clusters by Lynx Therapeutics, based on the mouse genome sequence (Release #3, Feb 2003) [45] and the mouse UniGene sequences (UniGene Build #122) [46]. Briefly, the mapping procedure included: extraction of 'virtual' signatures from genomic sequences, classification of 'virtual' signatures from genomic sequences, and matching of MPSS expressed signatures to genomic signatures [44]. For the comparison with microarray data, we included only the reliable signatures which were located closer to polyadenylation signal or poly(A) tail on a mRNA sequences with known orientation information [47]. If a UniGene cluster was found to be corresponding to multiple signatures in a given library, all tag counts were pooled to obtain the abundance of the UniGene cluster. If a tag was found to map to multiple UniGene clusters, the corresponding tag count was discarded.

### Gene mapping among microarray platforms and between microarray and MPSS

Two approaches to match probes across different microarray chips, annotation-based and sequence-based probe matching were used [6]. Briefly, by the annotation-based approach, we obtained UniGene (UG) and LocusLink (LL) based matching, whereas probe matches at the RefSeq (RS) and RefSeq-exon (RSEXON) levels by utilizing actual sequence information belong to the latter.

MPSS signatures were mapped to UniGene clusters, using an *in silico *constructed "virtual tags" library, as described above. Thus, the gene expression data measured by microarrays and by MPSS were paired up for comparisons via UniGene clusters.

### Biological variations and technical variations

Two separate total mRNA extraction processes were conducted on mouse retina under the same experimental settings and protocols, which generated mouse retina pool 1 (MRP1) and pool 2 (MRP2). Note that Illumina data were not included in this part of analyses due to lack of technical replicates.

#### Two-way ANOVA to identify individual genes of significant variation

To investigate the effects of biological variation and platform variation, two-way ANOVA (Analysis of Variance) was performed. As concluded in our previous study [6], the sequence-based cross-platform probe matching is more reliable than the annotation-based probe matching. Therefore, RS- and RSEXON-based mapping were used for this evaluation. For a given set of transcripts (RSs or RSEXONs) that were reliably detected in all chips of all platforms, the significances of sample-dependent bias, platform-dependent bias and interaction between sample and platform were determined for each transcript. Thus, we were able to observe the gene-specific effects of sample and platform biases.

#### SAM to characterize biological replicates' behavior in detecting expression changes

A group of differentially expressed genes represents the desired result of most microarray users. SAM (Significance Analysis of Microarray), proposed by Tusher *et al*., [48] is a method that can determine the significance of gene expression changes by permuting replicated measurements followed by an estimation of the false discovery rate (FDR). SAM assesses both the sensitivity and specificity of a microarray platform.

In our study, for each platform, the five pairs of chips on which MRP1 and MC were hybridized respectively were considered as one experiment group, while similarly another experiment group consisted of MRP2 and MC data. Prior to SAM analysis, the normalized log_2_ratios underwent two sequential filtering steps: (1) filtering according to spot quality flags; and (2) filtering out the probes which had less than three valid measurements out of the five replicates. For each microarray platform and each experiment group, the number of "called" genes (as differentially expressed) and FDR were recorded for every threshold "delta", which steps from 0.1 to 4 at an interval of 0.1.

#### GSEA to summarize differences between biological replicates using biological themes

GSEA (Gene Set Enrichment Analysis) [49,50] combines functional annotations and a statistical mechanism to determine a few sets of genes, each with a biological theme, which are over-/under-represented between two data sets representing two different classes. Instead of looking at per individual gene, GSEA is focused on gene sets pre-defined according to Gene Ontology (GO) category, pathway, or localization, etc, whose results have more robust and explicit biological interpretation. We used GSEA, a web-based application provided by Babelomics [51], to identify which biological process(es) and molecular function(s) were the most susceptible to the random differences between MRP1 and MRP2. The GSEA results of each platform were also compared.

#### One-way ANOVA analysis of microarray replicates and Z-test of MPSS runs

One-way ANOVA was conducted for each microarray platform (10 experiments: five for MRP1, five for MRP2). One-way ANOVA uses those genes that have valid measurements (filtered intensities after normalization) across all experiments as input, and assigns a p-value to each gene indicating whether this specific gene displayed significantly varied expression levels between two biological replicates.

MPSS analyses were also performed on the two pools of retina sample independently. Due to its high cost, it is not feasible to conduct repeated MPSS experiments for each biological sample. The common practice for those who use MPSS to identify differentially expressed genes has been to apply statistical tests that are able to handle non-replicate data based on certain sampling assumptions. The Z-statistics test [22] was applied to identify genes that were significantly differentially expressed between MRP1 and MRP2.

#### Accession numbers

The microarray data and MPSS data for the manuscript has been submitted to the GEO Omnibus. The series record number is GSE6313.

## Authors' contributions

FL designed and performed the data analysis workflow, drafted the manuscript. TKJ, EH, and WPK participated in the data analysis. WPK, JT and CP participated in running the microarray experiments. WPK, CC, LOM, and TKJ conceived the study and participated in the study design. All co-authors contributed to manuscript revisions and approved the final manuscript.

## Supplementary Material

Additional file 1 Evaluation of intra-platform and inter-platform data consistency (Affymetrix, Amersham, Mergen, ABI). This file shows the CVs and correlation coefficients of both the within-platform data set and among the across-platform data. Click here for file

Additional file 2 CAT (Correspondence At the Top) plots,. The CAT plots show cross-platform data correspondence when using one platform as the reference. Click here for file

Additional file 3 List of GO terms derived from GSEA (Gene Set Enrichment Analysis). This file provides a list of GO terms which are shown as being variable between the two biological replicate pools used in our study. Click here for file

Additional file 4 Illumina BeadArray^® ^Experimental Protocols. This is the detailed description of experimental protocols for Illumina BeadArray^® ^chip. Click here for file
