# Supplementary material for: Comparison of hybridization-based and sequencing-based gene expression technologies on biological replicates
Source: BMC Genomics. 2007 Jun 7;8:153. doi: 10.1186/1471-2164-8-153 (PMC1899500; doi:10.1186/1471-2164-8-153)
Supplement: Additional file 1 — Evaluation of intra-platform and inter-platform data consistency (Affymetrix, Amersham, Mergen, ABI). This file shows the CVs and correlation coefficients of both the within-platform data set and among the across-platform data. [file 1471-2164-8-153-S1.pdf]

Supplementary Material — A.

Evaluation of Intra-platform and Inter-platform Data Consistency (Affymetrix, Amersham, Mergen, ABI)

Table 1-a. Coefficient of Variations (CVs) of signal intensities across technical replicates within each platform. For each platform, CVs of all replicated measurements per probe were calculated. Table 1-a summarizes the means and standard deviations of the CVs of all probes in each platform.

Intra-Platform CVs of intensities (i.e. per sample)			MRP1::MC		MRP2::MC	
			mean	std	mean	std
Affy	Mouse Retina	raw	0.309	0.171	0.318	0.178
	Mouse Cortex		0.265	0.197	0.265	0.197
Amersham	Mouse Retina		0.196	0.132	0.228	0.152
	Mouse Cortex		0.175	0.138	0.175	0.138
Mergen	Mouse Retina		0.787	22.441	0.677	4.984
	Mouse Cortex		0.563	7.777	0.563	7.777
ABI	Mouse Retina		0.297	0.236	0.302	0.214
	Mouse Cortex		0.26	0.230	0.26	0.23
Affy	Mouse Retina	normalized_filtered	0.103	0.072	0.111	0.074
	Mouse Cortex		0.112	0.076	0.112	0.076
Amersham	Mouse Retina		0.101	0.072	0.125	0.076
	Mouse Cortex		0.103	0.063	0.103	0.063
Mergen	Mouse Retina		0.17	0.085	0.232	0.117
	Mouse Cortex		0.144	0.071	0.142	0.069
ABI	Mouse Retina		0.139	0.120	0.144	0.111
	Mouse Cortex		0.124	0.115	0.124	0.117

Table 1-b. Coefficient of Variations (CVs) of relative measurements (log_2ratios)across technical replicates within each platform. For each platform, CVs of all replicated measurements per probe were calculated. Table 1-b summarizes the means and standard deviations of CVs of all probes in each platform, in addition to the percentage of measurements having absolute log_2ratios lower than 1 or higher than 1.

Intra-Platform CVs of log2ratios			MRP1::MC			MRP2::MC		
			%	mean	std	%	mean	std
**Affymetrix**	**unfiltered**	**abs(l2r) <= 1 (*)**	0.787	0.492	0.441	0.777	0.513	0.448
		**abs(l2r) > 1**	0.213	0.341	0.350	0.223	0.359	0.347
**Amersham**		**abs(l2r) <= 1 (*)**	0.744	0.353	0.319	0.744	0.383	0.372
		**abs(l2r) > 1**	0.254	0.218	0.252	0.254	0.240	0.278
**Mergen**		**abs(l2r) <= 1 (*)**	0.700	0.666	0.557	0.679	0.694	0.563
		**abs(l2r) > 1**	0.297	0.426	0.441	0.317	0.462	0.452
**ABI**		**abs(l2r) <= 1 (*)**	0.738	0.559	0.429	0.733	0.556	0.419
		**abs(l2r) > 1**	0.262	0.296	0.307	0.267	0.295	0.297
**Affymetrix**	**filtered**	**abs(l2r) <= 1 (*)**	0.389	0.217	0.131	0.390	0.239	0.143
		**abs(l2r) > 1**	0.099	0.148	0.102	0.101	0.159	0.105
**Amersham**		**abs(l2r) <= 1 (*)**	0.533	0.220	0.142	0.523	0.235	0.139
		**abs(l2r) > 1**	0.168	0.151	0.126	0.165	0.156	0.107
**Mergen**		**abs(l2r) <= 1 (*)**	0.371	0.367	0.184	0.357	0.382	0.204
		**abs(l2r) > 1**	0.144	0.239	0.138	0.143	0.254	0.171
**ABI**		**abs(l2r) <= 1 (*)**	0.383	0.234	0.192	0.384	0.243	0.192
		**abs(l2r) > 1**	0.179	0.147	0.141	0.183	0.148	0.128

Table 1-c. Pearson and Spearman correlation coefficients of signal intensities among technical replicates within each platform. For each platform, there are five technical replicates per sample. Pearson and Spearman correlation coefficients were calculated for each possible pair of replicates, before and after filtering. The mean and standard deviation of correlation coefficients are reported in Table 1-c.

Intra-Platform correlation coefficients of log2ratios		MRP1::MC				MRP2::MC			
		Pearson		Spearman		Pearson		Spearman	
		mean	std	mean	std	mean	std	mean	std
Affymetrix	unfiltered	0.776	0.116	0.707	0.151	0.771	0.120	0.702	0.157
Amersham	unfiltered	0.888	0.058	0.836	0.084	0.872	0.073	0.821	0.095
Mergen	unfiltered	0.770	0.122	0.711	0.157	0.763	0.125	0.709	0.155
ABI	unfiltered	0.808	0.098	0.704	0.151	0.815	0.095	0.709	0.149
Affymetrix	filtered	0.947	0.029	0.910	0.049	0.935	0.042	0.894	0.068
Amersham	filtered	0.955	0.027	0.925	0.043	0.949	0.033	0.911	0.056
Mergen	filtered	0.906	0.074	0.856	0.112	0.897	0.064	0.852	0.089
ABI	filtered	0.967	0.017	0.946	0.028	0.965	0.019	0.943	0.032

Table 2-a. Pearson and Spearman correlation coefficients of $\log_2$ratios (normalized and filtered) across platforms were derived using RS(RefSeq)-, RSEXON (RefSeq-Exon)- based probe matching criteria. For each combination of probe matching approach, the upper triangle matrix corresponds to Pearson correlation coefficients, and the lower triangle section is Spearman correlations.

	MRP1::MC						MRP2::MC				
Inter-Platform correlation coefficients of log2ratios	Affymetrix	Amersham	Mergen	ABI	Illumina		Affymetrix	Amersham	Mergen	ABI	Illumina
**RS**											
**Affymetrix**	1	0.783	0.807	0.830	0.719		1	0.783	0.790	0.835	0.731
**Amersham**	0.739	1	0.767	0.806	0.703		0.739	1	0.751	0.807	0.716
**Mergen**	0.728	0.707	1	0.814	0.713		0.705	0.688	1	0.793	0.71
**ABI**	0.749	0.752	0.716	1	0.742		0.761	0.754	0.687	1	0.755
**Illumina**	0.65	0.634	0.611	0.621	1		0.677	0.654	0.618	0.647	1
**RS (Filtered)**											
**Affymetrix**	1	0.872	0.908	0.906	0.863		1	0.874	0.903	0.900	0.882
**Amersham**	0.857	1	0.852	0.864	0.826		0.859	1	0.853	0.863	0.835
**Mergen**	0.892	0.832	1	0.894	0.861		0.879	0.833	1	0.883	0.861
**ABI**	0.889	0.841	0.861	1	0.875		0.886	0.835	0.848	1	0.873
**Illumina**	0.827	0.794	0.821	0.832	1		0.857	0.814	0.839	0.858	1
**RSEXON**											
**Affymetrix**	1	0.806	0.826	0.847	0.733		1	0.808	0.805	0.853	0.74
**Amersham**	0.762	1	0.790	0.824	0.718		0.767	1	0.777	0.826	0.731
**Mergen**	0.744	0.734	1	0.845	0.715		0.717	0.715	1	0.822	0.706
**ABI**	0.770	0.762	0.731	1	0.754		0.786	0.767	0.696	1	0.764
**Illumina**	0.665	0.651	0.613	0.628	1		0.687	0.668	0.608	0.652	1
**RSEXON (Filtered)**											
**Affymetrix**	1	0.887	0.912	0.920	0.877		1	0.890	0.908	0.917	0.896
**Amersham**	0.881	1	0.866	0.875	0.844		0.887	1	0.865	0.880	0.85
**Mergen**	0.899	0.853	1	0.910	0.876		0.886	0.855	1	0.903	0.872
**ABI**	0.910	0.855	0.880	1	0.894		0.911	0.856	0.876	1	0.889
**Illumina**	0.845	0.821	0.836	0.86	1		0.873	0.841	0.85	0.884	1

Table 2-b. Inter-platform Pearson and Spearman correlation coefficients of intensities (filtered and percentile-transformed) were derived using UG(UniGene)-, RS(RefSeq)-, RSEXON (RefSeq-Exon)- based probe matching criteria. For each combination of probe matching approach, the upper triangle matrix corresponds to Pearson correlation coefficients, and the lower triangle section is Spearman correlaitons.

	MRP1						MRP2						MC				
Inter-platform Correlations of Percentile-transformed Intensities	Affymetrix	Amersham	Mergen	ABI	Illumina		Affymetrix	Amersham	Mergen	ABI	Illumina		Affymetrix	Amersham	Mergen	ABI	Illumina
**UG**																	
**Affymetrix**	1	0.634	0.583	0.645	0.523		1	0.626	0.577	0.649	0.528		1	0.619	0.589	0.638	0.520
**Amersham**	0.634	1	0.569	0.652	0.481		0.626	1	0.548	0.648	0.484		0.619	1	0.572	0.649	0.480
**Mergen**	0.585	0.572	1	0.611	0.497		0.579	0.551	1	0.603	0.490		0.591	0.574	1	0.614	0.511
**ABI**	0.656	0.672	0.613	1	0.570		0.657	0.665	0.602	1	0.579		0.649	0.669	0.618	1	0.566
**Illumina**	0.540	0.506	0.508	0.583	1		0.545	0.507	0.499	0.589	1		0.538	0.507	0.524	0.581	1
**UG (Filtered)**																	
**Affymetrix**	1	0.536	0.449	0.455	0.379		1	0.518	0.452	0.457	0.408		1	0.532	0.477	0.479	0.423
**Amersham**	0.548	1	0.481	0.562	0.450		0.528	1	0.460	0.557	0.445		0.544	1	0.497	0.577	0.472
**Mergen**	0.456	0.483	1	0.435	0.353		0.455	0.461	1	0.408	0.371		0.485	0.499	1	0.455	0.403
**ABI**	0.479	0.576	0.449	1	0.511		0.477	0.570	0.422	1	0.506		0.501	0.589	0.467	1	0.533
**Illumina**	0.402	0.460	0.362	0.513	1		0.429	0.457	0.378	0.506	1		0.450	0.487	0.413	0.535	1
**RS**																	
**Affymetrix**	1	0.659	0.639	0.696	0.487		1	0.652	0.631	0.701	0.501		1	0.652	0.651	0.698	0.494
**Amersham**	0.663	1	0.591	0.692	0.473		0.656	1	0.568	0.685	0.478		0.656	1	0.590	0.685	0.467
**Mergen**	0.643	0.593	1	0.657	0.503		0.636	0.571	1	0.648	0.498		0.655	0.593	1	0.669	0.517
**ABI**	0.712	0.723	0.666	1	0.603		0.713	0.713	0.654	1	0.608		0.716	0.716	0.679	1	0.602
**Illumina**	0.510	0.506	0.522	0.622	1		0.521	0.508	0.516	0.624	1		0.521	0.503	0.539	0.622	1
**RS (Filtered)**																	
**Affymetrix**	1	0.563	0.483	0.544	0.350		1	0.537	0.472	0.539	0.379		1	0.561	0.502	0.568	0.391
**Amersham**	0.572	1	0.487	0.621	0.442		0.545	1	0.460	0.613	0.430		0.569	1	0.490	0.628	0.459
**Mergen**	0.489	0.488	1	0.496	0.402		0.474	0.460	1	0.472	0.382		0.507	0.491	1	0.517	0.427
**ABI**	0.568	0.641	0.518	1	0.564		0.562	0.631	0.493	1	0.564		0.591	0.645	0.539	1	0.586
**Illumina**	0.372	0.456	0.412	0.568	1		0.393	0.444	0.394	0.567	1		0.413	0.478	0.447	0.589	1

**RSEXON**																	
**Affymetrix**	1	0.678	0.654	0.713	0.487		1	0.676	0.643	0.719	0.498		1	0.668	0.658	0.714	0.491
**Amersham**	0.682	1	0.586	0.716	0.473		0.680	1	0.568	0.712	0.476		0.674	1	0.590	0.700	0.462
**Mergen**	0.659	0.590	1	0.673	0.491		0.647	0.571	1	0.664	0.485		0.662	0.592	1	0.679	0.509
**ABI**	0.734	0.745	0.683	1	0.620		0.737	0.738	0.670	1	0.622		0.742	0.736	0.691	1	0.616
**Illumina**	0.536	0.513	0.523	0.644	1		0.544	0.514	0.515	0.642	1		0.550	0.511	0.544	0.640	1
**RSEXON (Filtered)**																	
**Affymetrix**	1	0.605	0.493	0.592	0.392		1	0.586	0.492	0.589	0.419		1	0.597	0.509	0.613	0.435
**Amersham**	0.615	1	0.466	0.651	0.460		0.596	1	0.446	0.647	0.449		0.606	1	0.463	0.658	0.479
**Mergen**	0.500	0.471	1.000	0.516	0.399		0.494	0.450	1	0.498	0.388		0.515	0.466	1	0.537	0.430
**ABI**	0.620	0.673	0.542	1	0.614		0.619	0.668	0.525	1	0.615		0.648	0.680	0.566	1	0.641
**Illumina**	0.446	0.479	0.426	0.626	1		0.460	0.472	0.414	0.624	1		0.490	0.504	0.466	0.648	1
