# Supplementary figures and images for: Comparison of hybridization-based and sequencing-based gene expression technologies on biological replicates
Source: BMC Genomics. 2007 Jun 7;8:153. doi: 10.1186/1471-2164-8-153 (PMC1899500; doi:10.1186/1471-2164-8-153)

**Supplementary Material — B**

**CAT (Correspondence At the Top) Plots**

A. MRP1

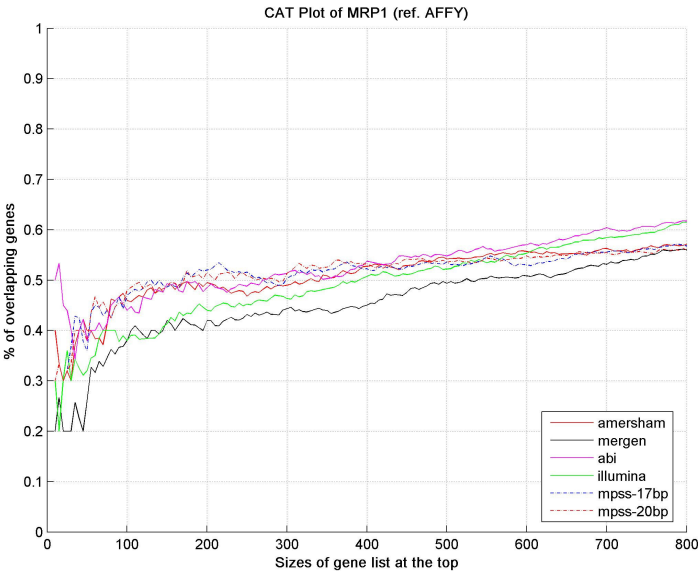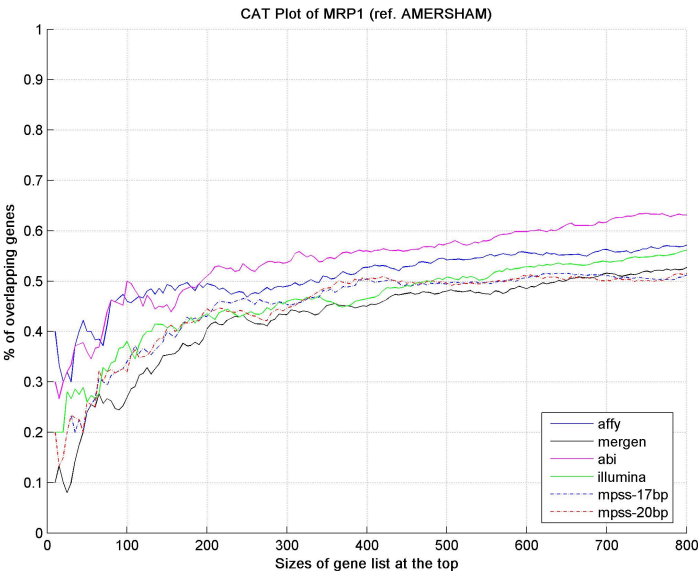

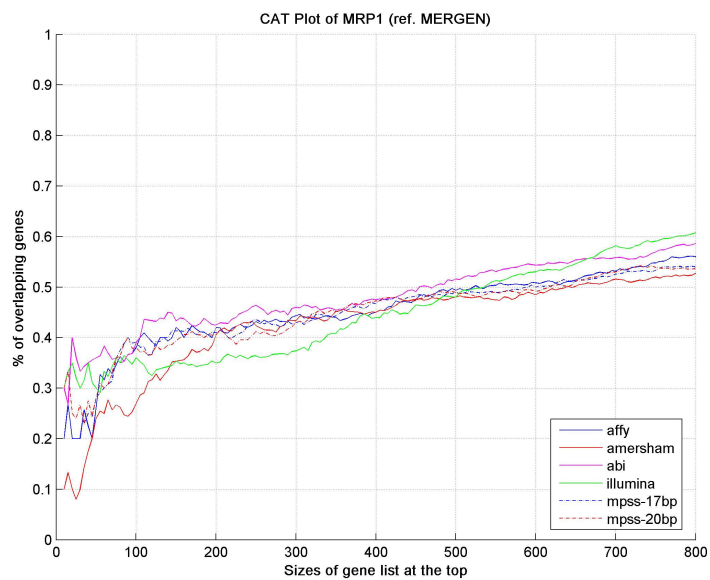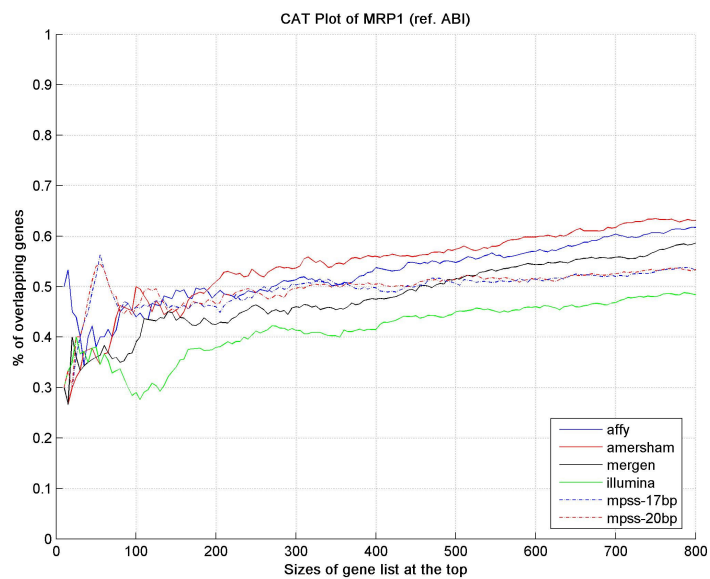

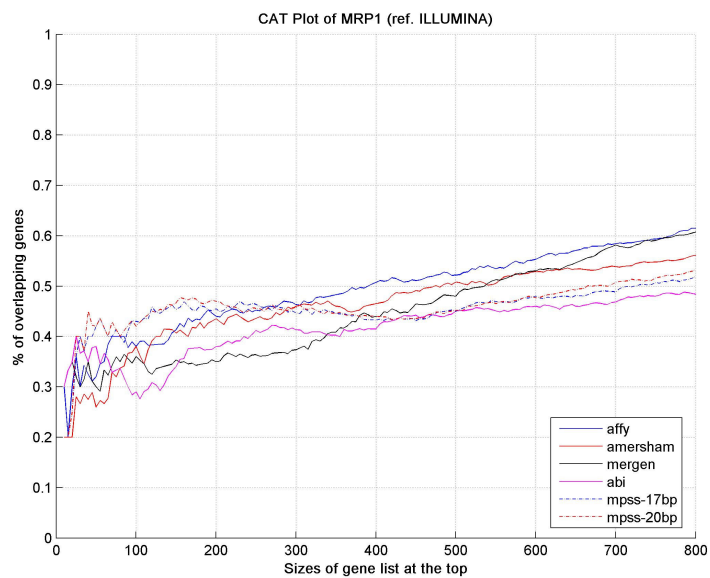

B. MRP2

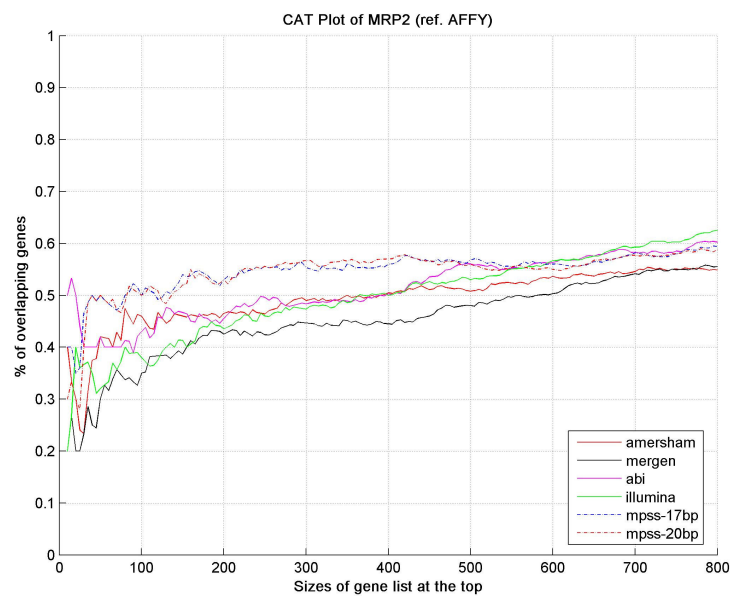

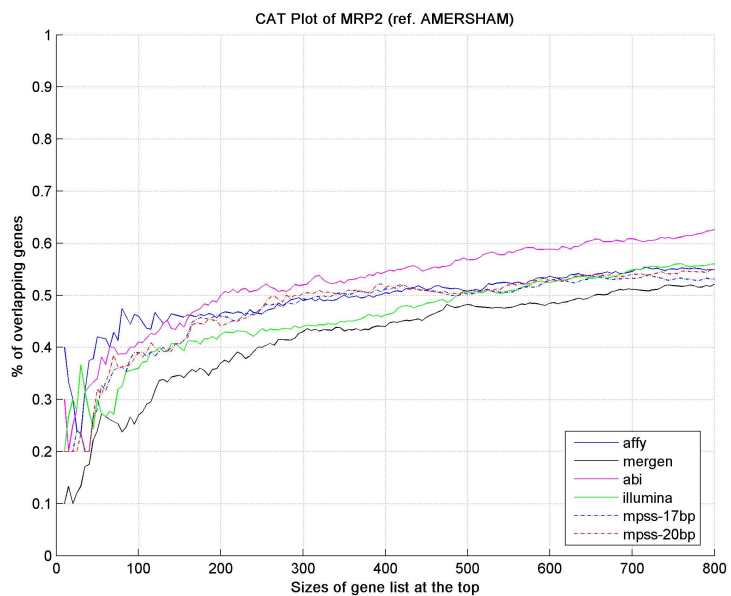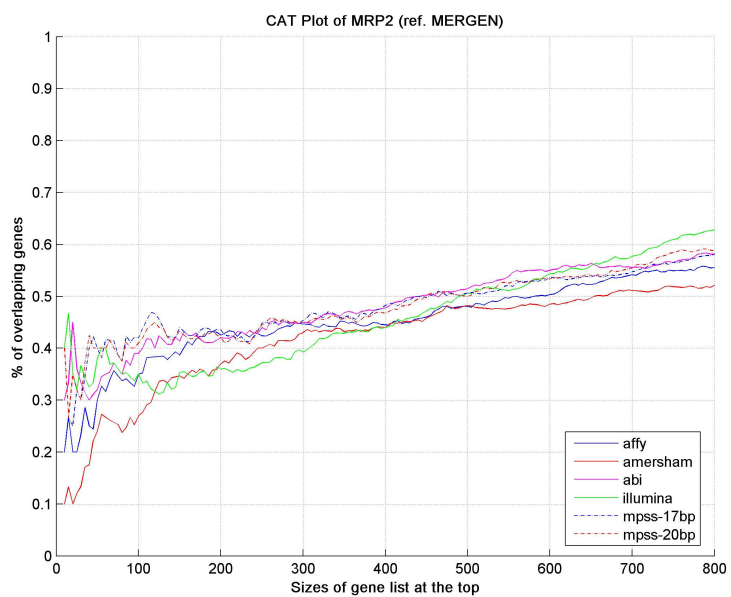

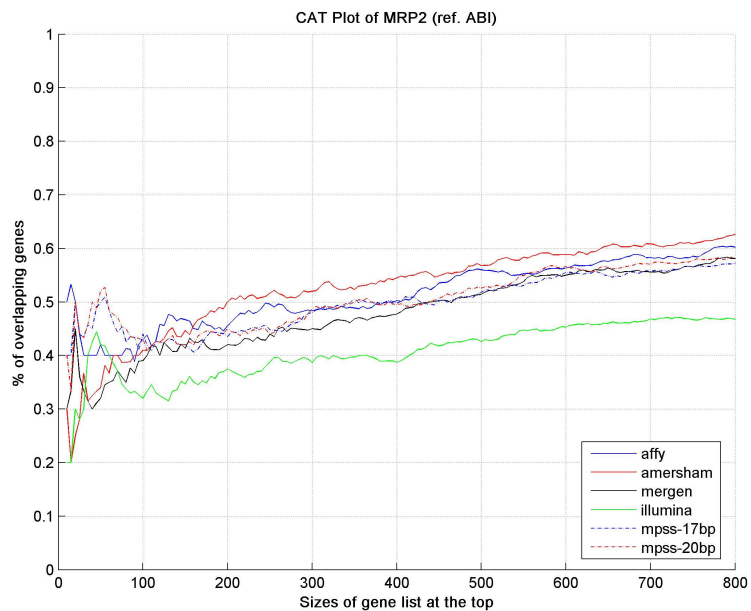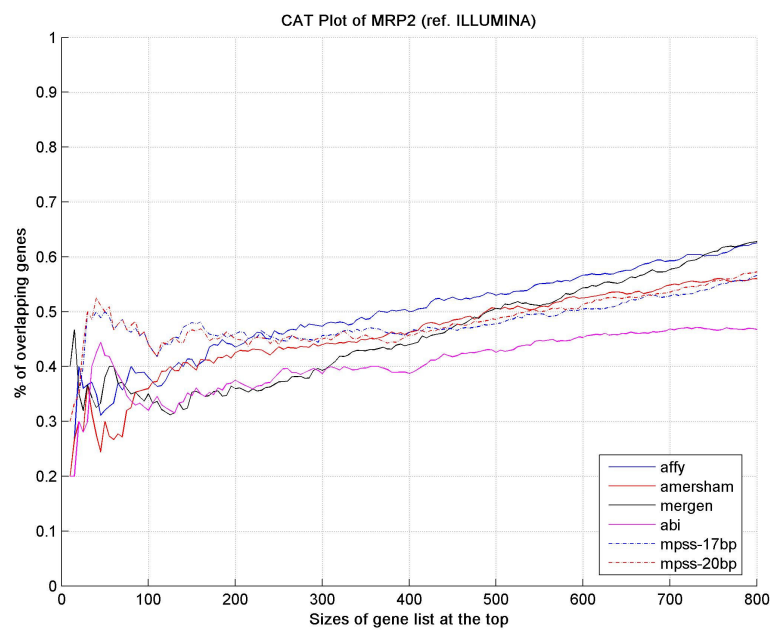

Supplement: Additional file 2 — CAT (Correspondence At the Top) plots,. The CAT plots show cross-platform data correspondence when using one platform as the reference. [file 1471-2164-8-153-S2.pdf]
