# Supplementary material for: Comparison of hybridization-based and sequencing-based gene expression technologies on biological replicates
Source: BMC Genomics. 2007 Jun 7;8:153. doi: 10.1186/1471-2164-8-153 (PMC1899500; doi:10.1186/1471-2164-8-153)
Supplement: Additional file 3 — List of GO terms derived from GSEA (Gene Set Enrichment Analysis). This file provides a list of GO terms which are shown as being variable between the two biological replicate pools used in our study. [file 1471-2164-8-153-S3.pdf]

***Supplementary Material — C***

## GSEA (Gene Set Enrichment Analysis) Results

A. List of 15 GO terms in ***Biological Process*** category that were enriched in MRP1 in comparison with MRP2, in all four platforms

Biosynthesis
Cell activation
Cell homeostasis
Cell motility
Cell proliferation
Cellular localization
Defense response
Hematopoietic or lymphoid organ development
Immune response
Localization of cell
Nitrogen component metabolism
Positive regulation of development
Protein localization
Regulation of transferase activity
Response to chemical stimulus

B. List of 12 GO terms in ***Molecular Function*** category that were enriched in MRP1 in comparison with MRP2, in all four platforms

ATPase activity (coupled to transmembrane movement of ions)
ATPase activity (coupled to transmembrane movement of substances)
Enzyme binding
Hydrolase activity (acting on acid anhydrides)
Oxidoreductase activity (acting on CH-OH group of donors)
Peptidase activity
Primary active transporter activity
Receptor binding
Transcription factor binding
Translation factor activity (nucleic acid binding)
Ubiquitin-like activating enzyme activity
Unfolded protein binding
