# Supplementary material for: Comparison of hybridization-based and sequencing-based gene expression technologies on biological replicates
Source: BMC Genomics. 2007 Jun 7;8:153. doi: 10.1186/1471-2164-8-153 (PMC1899500; doi:10.1186/1471-2164-8-153)
Supplement: Additional file 4 — Illumina BeadArray® Experimental Protocols. This is the detailed description of experimental protocols for Illumina BeadArray® chip. [file 1471-2164-8-153-S4.pdf]

Supplementary Material — D

Illumina BeadArray® Experimenal Protocols

I. Protocols for biotin-labeled cRNA target preparation

First and second strand cDNA synthesis is performed using the IlluminaAmp-kit (*Ambion*) using full volume RT and quarter-volume IVT reactions and biotin-labeling using Biotin-16-UTP (Perkin Elmer).

1. FIRST STRAND cDNA SYNTHESIS

1.1. a 1-to-10 dilution was required to bring the concentration under 100ng/ul. Pipette volume appropriate to 100 ng total RNA into appropriate well of a 8-tube strip tube. RNAse-free water was added to bring the volume to 11ul.

1.2 assemble First Strand RT Master mix in eppendorf tube

1.3.1 First Strand RT Master mix volume for 21 reactions (5 samples):

Reverse Transcription Master Mix

Assemble at room temp, adding reagents in the order shown

Component	per rxn	Reagent Amounts (µl) with 5% Extra
T7 Oligo(dT) Primer	1	22.1
10X First Strand Buffer	2	44.1
dNTP Mix	4	88.2
RNase Inhibitor	1	22.1
ArrayScript	1	22.1
**Total (µl)**	**9**	**198.6**

Use 9 µl of Reverse Transcription Master Mix per reaction.

1.3.2 mix and pulse centrifuge First Strand RT Master Mix tube

1.4 preheat MJ Tetrad to 42C with heated lid

1.5 pipette First Strand RT Master Mix

1.5.1 add 9 ul Master Mix to each well containing 11ul total RNA solution.

1.5.2 pipette mix samples in plate 10x

1.5.3 place caps on strip tubes

1.5.4 pulse centrifuge tubes

1.6 incubate samples at 42C for 2 hours

2. SECOND STRAND cDNA SYNTHESIS

2.1 place tubes on ice until ready to proceed with second strand synthesis
2.2 precool MJ Tetrad to 16C without heated lid. Be sure lid has cooled before further incubations
2.3 assemble Second Strand RT Master mix in eppendorf tube
2.3.1 Second Strand RT Master mix volume for 21 reactions (5 samples):

Second Strand Master Mix

Assemble on ice, adding reagents in the order shown

Component	per rxn	Reagent Amounts (µl) with 5% Extra
Nuclease-free Water	63	1389.2
10X Second Strand Buffer	10	220.5
dNTP Mix	4	88.2
DNA Polymerase	2	44.1
RNase H	1	22.1
**Total (µl)**	**80**	**1764.1**

Use 80 µl of Second Strand Master Mix per reaction.

2.3.2 mix Second Strand RT Master Mix tube.
2.4 pipette Second Strand RT Master Mix
1.5.1 add 80 ul Master Mix to each well.
1.5.2 pipette mix samples in plate 10x
1.5.3 place caps on tubes
1.5.4 pulse centrifuge tubes
2.5 incubate samples at 16C for 2 hours

3. PURIFICATION OF DOUBLE-STRANDED cDNA
3.1 prepare cDNA purification tubes
3.1.1 transfer 100 ul reaction to 1.5 ml eppendorf tube
3.1.2 add 250 ul cDNA Binding Buffer to cDNA and mix
3.1.3 add mix to a spin tube with membrane, being sure to look for bubbles on the membrane
3.2 centrifuge at 10,000 x g for 1 min. Discard contents after spin
3.3 add 500 ul of Wash Buffer (containing ethanol) to the double stranded cDNA on the membrane.
3.4 centrifuge at 10,000 x g for 1 min. Discard contents after spin
3.5 centrifuge at 10,000 x g for 1 final min to dry the column and get rid of residual wash buffer
3.6 for elution into provided collection microtubes, replace tubes with the provided collection microtube.
3.12 to elute, add 10 µl of RNase-free water at 55C to the center of each membrane, let stand for 2 min, and centrifuge at 10,000 x g for 1 min. Add an additional 10 µl of RNase-free water at 55C to the center of each membrane, let sit 1 min, and centrifuge at 10,000 x g for 1 min.

4. IN-VITRO TRANSCRIPTION

4.1 concentrate samples in SpeedVac

4.2 assemble IVT Master mix in eppendorf tube

4.2.1 IVT Master mix volume for 21 reactions (5 samples):

IVT Master Mix

Assemble at room temp, adding reagents in the order shown

Component	per rxn	Reagent Amounts (µl) with 5% Extra
Biotin-16-UTP	3.75	82.7
T7 ATP Soln	1	22.1
T7 CTP Soln	1	22.1
T7 GTP Soln	1	22.1
T7 UTP Soln	0.5	11
T7 10X Reaction Buffer	1	22.1
T7 Enzyme Mix	1	22.1
Nuclease-free Water	0.75	16.5
**Total (µl)**	**10**	**220.7**

Use 10 µl of IVT Master Mix per reaction.

4.2.2 mix and pulse centrifuge IVT Master mix tube.

4.3 preheat MJ Tetrad to 37C with heated lid

4.4 pipette IVT Master mix

4.4.1 add 10 ul Master Mix to each well containing dry cDNA.

4.4.2 pipette mix samples in plate 10x

4.4.3 place caps on tubes

4.4.4 pulse centrifuge

4.5 incubate samples at 37C for 16 hours 19 minutes.

Day 3: 5. cRNA PURIFICATION

5.1 add 90 ul RNase-free water to the 10 ul IVT reactions to adjust each sample volume to 100 µl.

5.2 transfer the samples to 1.5 ml eppendorf tubes

5.3 add 350 µl cRNA Binding Buffer to each sample, and mix by pipetting up and down 3 times.

5.4 add 250 µl of ethanol (96–100%) to each sample, and mix by pipetting up and down 3 times.

5.5. apply the samples from step 5.4 (700 µl) into the wells of the RNA Purification spin column.

5.6 centrifuge at 10,000 x g for 1 min. Discard contents after spin

5.7 add 650 ul of Wash Buffer (containing ethanol) to the double stranded cRNA on the membrane.

5.8 centrifuge at 10,000 x g for 1 min. Discard contents after spin
5.9 centrifuge at 10,000 x g for 1 final min to dry the column and get rid of residual wash buffer
5.10 for elution into provided collection microtubes, replace tubes with the provided collection microtube.
5.11 to elute, add 100 µl of RNase-free water at 55C to the center of each membrane, let stand for 2 min, and centrifuge at 10,000 x g for 1 min.
5.12 Store cRNA at –80°C. cRNA is now ready for quantification.

6. cRNA QUANTIFICATION

6.1 Prepare Ribogreen/TE working solution, to be stored with foil wrap in the dark. Use the Ribogreen within a few hours or remake from scratch.

- 6.1.1 Use the following formula to calculate the volume of Ribogreen needed. [# of samples + 10(std curve samples) x 0.55] = uL Ribogreen
- 6.1.2 Use the following formula to calculate the volume of TE needed: [[# of samples + 10 (std curve samples)] x 110] = uL TE
- 6.1.3 Mix the calculated volumes of TE and Ribogreen in 15 mL conical Falcon tube.
- 6.1.4 Vortex the tube until TE and RiboGreen are well mixed.
- 6.1.5 Store in dark until ready to use

6.2 Dilute the 100mg/mL RNA stock to 2ug/mL (50-fold)

- 6.2.1 Dispense 9800uL of 1X TE into 15 mL conical Falcon tube.
- 6.2.2 Add 200uL of 100mg/mL RNA stock to TE to make 10 ml of 2ug/mL RNA stock.
- 6.2.3 Vortex the RNA stock and 1X TE until well mixed.
- 6.2.4 Aliquot into 200 ul volumes to be used - one tube per standard curve.

6.3 Create standard curve RNA dilution for each plate as follows:

- 6.3.1 Mix and pulse centrifuge 2 ug/ml working stock RNA solution
- 6.3.2 Pipette these volumes into wells of the clear bottom plate:
  200 ng RNA = 0 ul 1X TE + 100 ul of 2 ug/ml Stock
  100 ng RNA = 50 ul 1X TE + 50 ul of 2 ug/ml Stock
  20 ng RNA = 90 ul 1X TE + 10 ul of 2 ug/ml Stock
  4 ng RNA = 98 ul 1X TE + 2 ul of 2 ug/ml Stock
  0 ng RNA = 100 ul 1X TE + 0 ul of 2 ug/ml Stock
- 6.3.3 Dispense standard curve into wells

6.4 If necessary, dilute unknowns to an approximate concentration of 100ng/uL
6.5 Dispense 99uL of 1X TE into each UV well that will hold a sample
6.6 Dispense 1uL of sample into each well containing 1X TE
6.7 To each well containing sample or standard curve add 100uL RiboGreen working solution.
6.8 Cover UV plate with foil and let stand 2 to 5 minutes, protected from light.
6.9 Set the Molecular Devices microplate fluorometer as follows:
Excitation – 485nm
Emission – 535nm

6.10 Using the Mol Devices microplate fluorometer, measure fluorescence.
6.11 Generate Standard Curve from known concentrations by using RNA_Quantitation excel template.
6.12 Using linear equation for the Standard Curve, apply formula to unknown samples.

Material

ddH$_2$O; RNase-free
IlluminaAmp kit; (*Ambion*) containing
Biotin-16-UTP (*Perkin Elmer*)
Strip tubes and caps
Eppendorf Tubes
100% Ethanol
RiboGreen Kit (Molecular Probes)
  Includes:
  100 ug/ml E.coli RNA Stock solution
  RNA dye concentrate
RNAse Zap
20X TE
Aluminum Foil

Equipment

SpeedVac 110 (*Thermo Savant*)
Eppendorf centrifuge
Plate Centrifuge
MJ Tetrad
Molecular Devices SpectraMax Plate Fluorometer

II. Protocols for SAM Hybridization experiment

1. Hybridization Plate Setup
    1.1. One microgram of cRNA from each sample is aliquoted into the 384 well hybridization plate.
    1.2. Pipettete the volume into alternating wells (A1, C1, …A3, C3…) using the blue template.
    1.3. Dry the cRNA down in the SpeedVac without heat because variable volumes of cRNA are required to aliquot 1 ug. Do not overdry.

2. Mix with Hybridization Reagents
    2.1. Add 13.3 ul RNase-free water to every sample well.
    2.2. Pipettete up and down 10-15x
    2.3. Cover with a plastic film seal and let sit 10 minutes at 4C.
    2.4. Prepare Hybridization Mix
        2.4.1. In a 15 ml Falcon tube, add 1760 µL Hyb E1 buffer with1060 µL formamide. Mix well by vortexing
    2.5. Dispense 26.7 µL hybridization mix into wells containing cRNA and water. Mix well.
    2.6. Prepare Humidity Control Mix
        2.6.1. In a 15 mL Falcon tube, mix 3.75 mL HC E1 buffer with 1.25 mL formamide. Mix well by vortexing.
    2.7. Dispense 40 µL Humidity Control mix into alternate wells (B2, B4…D2, D4…) using the humidity control template between wells containing cRNA and hyb solution.

3. Hybridize to SAM
    3.1. Pulse centrifuge hyb plate at 2000 x g
    3.2. Insert left edge of HYB plate into the inside left recessed area of the SAM alignment fixture
    3.3. Using two hands, carefully insert the right edge of the HYB plate into the inside right recessed area of the SAM alignment fixture.
    3.4. Verify proper seating
    3.5. Apply a red HYB plate perimeter gasket to the HYB plate top perimeter. Ensure no wells are covered with the gasket
    3.6. Open the mylar pouch containing the SAM (done just before the hyb is set up)
    3.7. Align and seat the SAM into the SAM alignment fixture, using the SAM outer skirt alignment guides
    3.8. Carefully lower the SAM into position, bundle side down, in the fixture
    3.9. Position red proximal end gasket on top of SAM so that bundle ends align with gasket holes
    3.10. Place black metal top plate on top of proximal end gasket and into SAM alignment fixture top recesses, metal feet pointing upward.
    3.11. Place SAM alignment fixture clamps onto long sides of the fixture (two clamps per side), securing metal top plate (on top) and HYB plate (on the bottom)

3.12. Inspect bottom of HYB plate to verify that SAM bundles are centered in the plate wells. Do Not Invert. If not centered, disassemble and repeat.
3.13. Ensure the oven is set to 55°C.
3.14. Incubate overnight (16 hours).

4. Set up Wash
   4.1. Set up First Wash E1 Buffer Plate
      4.1.1. Pour approximately 5 mL Wash E1 buffer into a solution trough.
      4.1.2. Using an 12-channel precision pipette, dispense 40 µL Wash E1 buffer into each well of odd columns, every other row of the 384-well WASH plate
      4.1.3. Add adhesive seal
      4.1.4. Pulse centrifuge plate at 2000 x g
   4.2. Set up Second Wash E1 Buffer Plate
      4.2.1. Pour approximately 5 mL Wash E1 buffer into a solution trough.
      4.2.2. Using an 12-channel precision pipette, dispense 40 µL Wash E1 buffer into each well of odd columns, every other row of the 384-well WASH plate
      4.2.3. Add adhesive seal
      4.2.4. Pulse centrifuge plate at 2000 x g
   4.3. Set up Preblock Casein Plate
      4.3.1. Pour approximately 5 mL Blocker Casein in PBS buffer into a solution trough.
      4.3.2. Using an 12-channel precision pipette, dispense 40 µL Blocker Casein in PBS buffer into each well of odd columns, every other row of the 384-well WASH plate
      4.3.3. Add adhesive seal
      4.3.4. Pulse centrifuge plate at 2000 x g
   4.4. Set up Signal Detection Plate
      4.4.1. Resuspend SA-Cy3 powder (1 mg) with 1 mL RNase-free water. Store in 50 ul aliquots at -20C.
      4.4.2. Dispense 5000 µL Blocker Casein in PBS into a 15 mL Falcon tube.
      4.4.3. Add 5 ul SA-Cy3 into the 15 mL Falcon tube. Vortex thoroughly
      4.4.4. Pour SA-Cy3/Casein solution into a solution trough.
      4.4.5. Using an 12-channel precision pipette, dispense 40 µL SA-Cy3/Casein in PBS buffer into each well of odd columns, every other row of the 384-well WASH plate
      4.4.6. Add adhesive seal. Pulse centrifuge plate at 2000 x g
      4.4.7. Store any unused SA-Cy3, protected from light, at 4°C until needed again. Do not refreeze unused SA-Cy3.
   4.5. Set up Third Wash E1 Buffer Plate
      4.5.1. Pour approximately 5 mL Wash E1 buffer into a solution trough.
      4.5.2. Using an 12-channel precision pipette, dispense 40 µL Wash E1 buffer into each well of odd columns, every other row of the 384-well WASH plate
      4.5.3. Add adhesive seal
      4.5.4. Pulse centrifuge plate at 2000 x g
5. Wash

5.1. When incubation period is complete, remove the HYB plate/SAM pair from the oven.
5.2. Remove clips, top plate, and proximal end gasket from HYB plate/SAM pair
5.3. First Wash – (5 Minutes)
    5.3.1. Quickly remove the SAM from the HYB plate. Do not let the SAM dry out.
    5.3.2. Use the second SAM alignment fixture to align the SAM with the first wash plate.
    5.3.3. Immediately place the SAM in the first Wash E1 buffer plate, and let stand for 5 minutes at room temperature.
5.4. Second Wash – (10 Minutes)
    5.4.1. Quickly remove the SAM from the first Wash E1 buffer plate.
    5.4.2. Use the second SAM alignment fixture to align the SAM with the second Wash E1 buffer plate.
    5.4.3. Immediately place the SAM in the second Wash E1 buffer plate, and let stand for 10 minutes at room temperature.
5.5. PreBlock Casein – (5 Minutes)
    5.5.1. Remove the SAM from the second Wash E1 buffer plate.
    5.5.2. Use the second SAM alignment fixture to align the SAM with the preblock wash plate.
    5.5.3. Transfer the SAM to the preblock wash plate, and let stand for 5minutes in Blocker Casein in PBS.
5.6. Signal Detection – (10 Minutes)
    5.6.1. Remove the SAM from the preblock wash plate.
    5.6.2. Use the second SAM alignment fixture to align the SAM with the signal detection plate.
    5.6.3. Transfer the SAM to the signal detection plate, and let stand for 10 minutes in SA-Cy3/ Casein solution.
5.7. Third Wash – (5 Minutes)
    5.7.1. Quickly remove the SAM from the signal detection plate.
    5.7.2. Use the second SAM alignment fixture to align the SAM with the third Wash E1 buffer plate.
    5.7.3. Immediately place the SAM in the third Wash E1 buffer plate, and let stand for 5 minutes at room temperature.
5.8. Dry Bundles
    5.8.1. Holding the Whoosh-Duster canned air approximately 1-2 cm away from the SAM, dry the SAM. Dry each bundle individually.
    5.8.2. If Clean Lab Air or Nitrogen is available, follow the same procedure to dry each bundle

6. Proceed to Image SAM.

<u>Material</u>
384 well plates -Thermo Labsystems, catalog # 9504000
Customer RNA Samples

RNase-free water
15 ml Falcon tubes
Hyb E1 buffer
HC E1 buffer
Deionized Formamide
Wash E1 Buffer
Blocker Casein in PBS buffer
SA-Cy3 Stock solution (1 mg/ml)
Adhesive plate seal
Foil plate seal
Whoosh duster canned air or clean lab Air/Nitrogen with nozzle

<u>Equipment</u>
SpeedVac (e.g. SpeedVac 100 Thermo Savant)
Plate Centrifuge
SAM alignment fixture
Hyb assembly
- Hyb plate perimeter gasket
- Hyb proximal end gasket
- Top plate
- 4 Binder clips

Hyb Oven

III. Protocols for Scanning of Illumina SAM Hybridizations using SentrixScan Software

1. SAM Scan
   1.1. Load Array Matrix
      1.1.1. Start the Scanner
         1.1.1.1. Power up the BeadArray Reader 30 minutes before proposed scan (On switch on the back panel)
         1.1.1.2. Power up the BeadArray Reader computer
         1.1.1.3. Double click the SentrixScan icon on the desktop to start the SentrixScan software
         1.1.1.4.If there is no adaptor tray, load **blue** adaptor tray for GEX SAMs, using the three orientation holes as guides.
      1.1.2. Using the hand-held barcode scanner, scan the barcode of the SAM before loading it into the adaptor tray, as the barcode will be face down when loaded
      1.1.3. To load SAM, hold it by the outer edges of the shorter side and place it onto the adaptor tray, aligning the three alignment balls correctly
      1.1.4. Using the hand-held barcode scanner, scan the SAM barcode position
      1.1.5. Click the Close Tray button at the bottom of the screen to load the SAM into the scanner
   1.2. Acquire Image
      1.2.1. Connect to the Scanner by Right or Left clicking on the Illumina logo. Under the Scanner menu, select Connect.
      1.2.2. Initialize the scan by Right or Left clicking on the Illumina logo. Under the Scanner menu, select Initialize.
      1.2.3. Click the Scan button and the Setup screen displays.
      1.2.4. Set SAM properties – Setup screen shows SAM Layout
         1.2.4.1. Confirm barcode for SAM is correct, or enter the corresponding SAM barcode into the Serial # box.
         1.2.4.2. To choose a Sentrix Type, click the Browse for Sentrix Type button. Click Predefined Sentrix Type and select Array Matrix for Sentrix Type
         1.2.4.3. In the Scan Settings box, choose Std GX for predifined PMT and Gain settings or Custom to choose your own PMT and Gain settings.
         1.2.4.4. To choose Data Repository and Decode Map locations, along with other scanner settings, click the Edit... button on the Setup screen. The Settings dialog displays. Make desired changes and click OK.
      1.2.5. Select sections
         1.2.5.1. Once you have set the properties for the Array, the Section Selection window displays
         1.2.5.2. Select or deselect individual array sections, click on the section to toggle it on or off. If all sections all sections are not selected, a warning will be generated when you begin the scan.

1.2.5.3. To select or deselect all of the sections in an area, click and drag over the sections to toggle them on or off.

1.2.5.4. To select or deselect an entire row of sections, click and drag over the numbers or letters.

1.2.6. After selecting the array sections to scan, click Scan to start the scan. The SentrixScan application will automatically tilt and align the array for the scanning process.

1.2.7. The blue lights on the front panel of the scanner will illuminate indicating the scan is underway. A full scan takes about 90 minutes. The first 5-6 minutes represent the Reader's internal focusing, de-tilting, and image centering process, and require no operator interaction.

1.2.8. When you begin a scan, the Preview window automatically displays the array being scanned, providing feedback as the scan progresses. You can view different sections of the array while it is being scanned by clicking and dragging the yellow box in the preview window over the desired viewing section.

1.2.9. The SentrixScan software provides a number of options for controlling the scan, allowing you to start, pause, or stop the scan. You can also control the preview window that provides visual feedback of the scan.

1.2.9.1. While on the Scan screen, click the Pause button to pause the scan

1.2.9.2. While on the Scan screen, click the Resume button to resume the scan

1.2.9.3. While on the Scan screen, click the Stop button to stop the scan

1.2.9.4. While on the Scan screen, click the Start button to restart a stopped scan

1.2.10. If any sections did not scan correctly, could not be registered, or did not meet intensity threshold values, the Failed Sections dialog box is displayed at the end of the scan. To rescan failed sections, place a check mark in front of the sections to rescan and click Rescan Selected.

1.2.11. To Open and View an Array after the scan, click on a scanned section from the Status Window. The image displays in the Preview Window.

1.2.12. By right clicking in the Image Window you can:

1.2.12.1. Auto Contrast

1.2.12.2. Show Cores

1.2.12.3. Copy to Clipboard

1.3. SentrixScan software automatically registers and extracts image(s)

1.4. If registration or analysis fails, rescan or rerun analysis in AnEx software. Ensure the .ebm file correctly matches the BeadChip barcode or alignment will fail.

1.5. Click the bottom Load button on the scanner to eject/open the tray door and remove the SAM from the tray.

1.6. Scan the next SAM, or close the tray door using the close tray door button at the bottom of the page of the interface

<u>Material</u>

Customer SAM

<u>Equipment</u>

BeadArray Reader (Sherlock) with Blue Adapter for Bead-End reads
Blue slide tray for Blue adapter
Computer and SentrixScan Software with Storage Server
SAM CD with Array Content/Bead Manifest and Decode Data
